# Antioxidant Properties of Maqui Berry Extract (*Aristotelia chilensis* (Mol.) Stuntz) and Its Potential Photoprotective Role on Human Skin Fibroblasts

**DOI:** 10.3390/molecules28237802

**Published:** 2023-11-27

**Authors:** Marta Wacewicz-Muczyńska, Justyna Moskwa, Anna Puścion-Jakubik, Sylwia K. Naliwajko, Marek Niczyporuk, Katarzyna Socha

**Affiliations:** 1 Department of Specialist Cosmetology, Medical University of Bialystok, Akademicka 3 St., 15-267 Bialystok, Poland; 2 Department of Bromatology, Medical University of Bialystok, Mickiewicza 2D St., 15-222 Bialystok, Poland; justyna.moskwa@umb.edu.pl (J.M.); anna.puscion-jakubik@umb.edu.pl (A.P.-J.); sylwia.naliwajko@umb.edu.pl (S.K.N.); katarzyna.socha@umb.edu.pl (K.S.); 3 Department of Aesthetic Medicine, Medical University of Bialystok, Akademicka 3 St., 15-267 Bialystok, Poland; marek.niczyporuk@umb.edu.pl

**Keywords:** maqui, total phenolic content, antioxidant activity, photoprotector, fibroblasts, UVB radiation

## Abstract

Maqui berry (*Aristotelia chilensis*) is characterized by antioxidant and anti-inflammatory properties. The aim of this study was to evaluate the effect of maqui berry extracts on human skin fibroblasts (NHSFs) exposed to ultraviolet radiation (UVB). The photoprotective properties of the extracts were investigated via the determination of the total polyphenolic content (TPC) and antioxidant capacity (DPPH), and the chemical composition was assessed. The chemical purity of the extracts was studied via the evaluation of the toxic elements level. The water extract (MWE 57.75 ± 0.44 mg GAE/g) had the highest mean polyphenol content. The water (MWE) and ethanol (MEE70) extracts had the highest inhibitory activities against DPPH radical formation (283.63 ± 7.29 and 284.60 ± 4.31 mg Tx/L, respectively). The analyzed extracts were found to be safe in terms of toxic elements (arsenic, cadmium, lead). The tested extracts of maqui berry did not cause a cytotoxic effect on NHSF cells after 24, 48, and 72 h of incubation. When the NHSF cells were exposed to UVB radiation in the presence of maqui extracts, their viability was increased or maintained. The maqui berry extracts had a slightly protective effect against skin damage caused by UVB radiation. These were preliminary studies that require further research to determine which maqui compounds correspond with the photoprotective activity.

## 1. Introduction

Maqui (*Aristotelia chilensis* (Mol.) Stuntz) is a native evergreen shrub that mainly grows in central and southern America, especially Chile and Argentina. It is a plant of the *Elaeocarpaceae* family. Its purple-black berries are known worldwide for their extraordinary antioxidant properties found mainly in the fruit [1,2]. In comparison with other berries, maqui has significant amounts of flavonoid antioxidants and bioactive properties [3]. Maqui is considered to be one of the healthiest fruits because it is an extremely rich source of bioactive compounds, such as flavonoids, tannins, phenolic acids, stilbenes, and anthocyanins [4]. It was reported that the main identified phenolics were gallic, protocatechuic, and ellagic acids, and the main free flavonoids identified in maqui berries were myricetin and quercetin [5,6]. Several authors reported the presence of eight anthocyanins in maqui berries, which were identified as delphinidin or cyanidin-derivative. Delphinidin derivatives were the predominant anthocyanins detected, with delphinidin-3-glucoside as the main component, followed by delphinidin-3,5-diglucoside and delphinidin-3-sambuboside [5,7,8].

Several health benefits and bioactivities of maqui berry extracts have been reported, e.g., the inhibition of low-density lipoprotein oxidation, antiphotoaging of the skin [1,9], antimicrobial and anticarcinogenic effects, antihemolytic protection, prevention from atherosclerosis, cardio-protection, obesity control, and inhibition of adipogenesis and diabetes symptoms [10,11,12,13,14].

Recent studies conducted on the biological activities of maqui berry demonstrated strong antioxidant [15,16] and anti-inflammatory properties [9,12]. Maqui is known to be capable of oxygen radical absorbance, xanthine oxidase inhibition, and intracellular oxidative stress reduction. The antioxidant activity is mainly due to the polyphenolic fraction, specifically anthocyanin-based compounds that account for over 80% of the total polyphenols [17,18]. Among the polyphenols, anthocyanins were shown to have powerful antioxidant and anti-inflammatory effects [19,20]. There have been various extraction methods that are usually applied for covering phenolic compounds, such as conventional method extraction, microwave, pulse electric field, vacuum-cold plasma, and enzymatic pretreatment. A recent study showed that enzymatic (pectinase) pretreatment resulted in the highest extraction of anthocyanins and polyphenols [21].

It is well known that exposure to solar UV radiation, particularly its UVB component, causes many adverse effects on the skin, including photoaging, inflammation, photoimmunosuppression, and photocarcinogenesis [22]. Previous experiments showed that maqui berry can suppress light-induced photoreceptor cell death by inhibiting ROS (reactive oxygen species) generation, suppressing the LPS-induced production of NO (nitric oxide), resisting oxidative stress, and might be an effective therapeutic agent protecting against skin damage caused by UVB radiation [23,24]. Several studies have reported potential benefits of the oral administration or topical applications of various polyphenols, which prevented or treated skin conditions (e.g., skin photo-damage) in animals and humans. Due to their anti-inflammatory properties and photo-chemopreventive effects (such as DNA repair and antioxidant activities), polyphenols were suggested to play an alternative role or enhance sunscreens [25]. Limited scientific reports have indicated the beneficial effects of maqui berry formulations on skin; however, their role as a photoprotective agent remains unclear.

To the best of our knowledge, there has been only one study conducted on the photoprotective properties of maqui berry extract, which were determined using human keratinocytes. Due to this, we performed a study whose purpose was to examine the potential photoprotective effects of maqui berry extracts on human skin fibroblasts exposed to UV irradiation in in vitro studies. Moreover, the quality assessment of maqui berry extracts was performed via the determination of total polyphenolic content (TPC), antioxidant capacity (DPPH assay), toxic elements (As, Pb, Cd) content, and the examination of the chemical composition.

## 2. Results and Discussion

### 2.1. Total Phenolic and Antioxidant Activities

The antioxidant activities of maqui extracts are directly correlated with the phenolic content. Maqui berries are known for the presence of compounds such as phenolic acids, flavonoids, and anthocyanins. The differences in the contents of bioactive ingredients in maqui berries can be attributed to various factors such as the environment, genotype and maturity stage of the fruits, and storage conditions [5,26]. Regardless of the various conditions and the type of drying (freeze, sun, vacuum drying), it was found that the final product of maqui berry was characterized by a high level of phenolic compounds [27].

The results of the TPC determination of the extracts tested in this work are shown in Table 1. In the case of maqui berry extracts, the water extract (MWE, 57.75 ± 0.44 mg GAE/g) had the highest mean polyphenol content. The TPC contents in the 70% ethanol extract (MEE70) and 95% ethanol extract (MEE95) were similar (56.47 ± 0.19 mg GAE/g and 56.65 ± 0.69 mg GAE/g, respectively). In previous studies, various phenolic contents of maqui berries were reported. Rubilar et al. [2] investigated crude extracts from fresh berries and showed a similar level to the one we found in our research, namely, a TPC of 45.7 mg GAE/g. Genkowsky et al. [5] also showed a high polyphenolic content of 49.74 ± 0.57 g GAE/kg. In the study performed by Sobaszek et al. [27], the TPC of maqui freeze-dried powder that was used for gel formation was characterized by 34.82 mg GAE/g. The study performed by Issis et al. [28] showed that TPC ranged from 23.16 to 36.49 mg GAE/g depending on the various temperatures used in the vacuum-drying process. Dara et al. [21] showed that the enzymatic pretreatment resulted in the highest extraction of anthocyanins (279.64 mg/L) and polyphenols (484.93 mg/L) from *Berberis vulgaris L*. Authors compared the results to the values of polyphenols and anthocyanins extracted using a pulsed electric field and vacuum-cold plasma.

The results obtained by Fredes et al. [7] show significant differences between different genotypes and between different geographical regions (10.7–20.5 mg GAE/g fresh weight). These results are consistent with others: Gonzalez et al. [26], who showed TPC levels of 21–28 mg GAE/g dried weight, and Brauch et al. [8], who reported that fresh and dry maqui berries (Patagonia region) were characterized by TPCs of 19.7 and 32.0 g GAE/kg, respectively. In another study, Fredes et al. [29] determined that maqui berries had the highest TPC (14.6 g GAE/kg) compared with fruits from the same region, including raspberries, blackberries, and strawberries, which also have a well-documented phenolic content. These results are consistent with previous studies [14,30], where maqui berries were compared with other polyphenol-rich fruits.

Maqui berries are considered a good source of antioxidant compounds due to showing high activity with respect to the DPPH decoloration assay. The antioxidant potential of maqui berries has been reported.

As shown in Table 1, the water (MWE) and ethanol (MEE70) extracts had the highest inhibitory activity against DPPH radical formation (283.63 ± 7.29 and 284.60 ± 4.31 mg Tx/L, respectively). Much lower values were attained by MEE95 (211.80 ± 6.14 mg Tx/L). Moreover, the MWE and MEE70 extracts exhibited a concentration dependence in their DPPH radical scavenging activities: the highest activities were shown by the MWE extract (66.04% inhibition) and MEE70 (64.35%). Our results are consistent with the study performed by Nowak et al. [31], who conducted a test using the DPPH in maqui juices and reported that it was 252 ± 3.0 mg Tx/L. In various studies, the free radical scavenging activity demonstrated in the DPPH test showed promising antioxidant properties of maqui berry of various study materials, including fresh fruits, powder, and extracts. Genkowsky et al. [5] reported that the DPPH value was 28.18 ± 0.37 g TE/kg. The results performed by Cespedes et al. [19], Fredes et al. [7], and Sobaszek et al. [27] show IC50 values for the DPPH assay of 0.0016 g/L, 0.0012 g/L, and 3.38 mg/mL, respectively. Rivera-Tovar et al. [9] also reported high radical scavenging capacity using the EC50 method (0.17–0.37 g dry extract/g DPPH). Lopez DiCastillo et al. [32] investigated the antioxidant activity of maqui in different solvent systems, including pure ethanol, ethanol 50%, and pure water, and showed significant differences between the DPPH values, with 122 ± 3, 128 ± 2, and 98 ± 1 mg Trolox/g fruit, respectively. Issis et al. [28] reported a high capacity of DPPH in fresh fruits (240.29 ± 6.23 µmol TE/g) and showed that a temperature of 80 °C is suitable for the vacuum drying of maqui berries because at 50–70 °C, the antioxidant capacity of DPPH decreased. Bastias-Montes et al. [33] indicated that the loss of antioxidant activity is lower compared with the control when the drying inlet temperature increases. They found that the DPPH activity was 93.38% of the control extract (initial extract), 70% of drying at 130 °C, and 85% of drying at 170 °C. According to the antioxidant activity, the best drying condition is the treatment at 170 °C.

### 2.2. Chemical Composition and Toxic Element Levels

GC-MS is one of the most reliable biophysical methods due to its specificity and repeatability. To the best of our knowledge, there have been only a few studies that analyzed the composition of maqui berry using gas chromatography–mass spectrometry (GC-MS).

In this study, the chemical composition of MME was investigated. GC-MS screening in the electron impact mode (EI) identified about 51 compounds. A list of these constituents and classification into specific groups of compounds is presented in Table 2 and Table 3. Unsaturated (34.77%) and saturated (23.18%) fatty acids and esters were the main components of the MME. However, previous authors found a higher contribution of each group of fatty acids. Brauch et al. [8] reported that the total unsaturated lipid fraction from maqui was found to be highly unsaturated up to 86% (including MUFAs up to 32% and PUFAs up to 53%), and the saturated fatty acid (SFA) content was found to be up to 15%. The PUFA/SFA ratios ranged from 3.2 to 3.5 depending on the maqui samples, including fresh juice, pomace, or dried. These results correspond with the results reported by Quispe-Fuentes et al. [6], who observed that the lipid fraction from maqui was highly unsaturated (up to 83%), with high PUFA/SFA ratios ranging from 3.24 to 3.50 (according to sample form-fresh or dried maqui fruits). Increased consumption of foods with a high PUFA/SFA ratio is associated with a lower risk of coronary heart disease.

In this work, the major ingredients of unsaturated fatty acids and esters were oleic acid (14.2%), 9,12-Octadecadienoic acid (7.5%), linoleic acid-ethyl ester (4.3%), oleic acid- ethyl ester (3.83%), and others (Table 2). The essential compounds from the group of saturated acids and esters were palmitic acid (14.9%), nonanoic acid (2.5%), hexadecanoic acid-ethyl ester (2.0%), and stearic acid (1.75%). Other compounds identified in this study of MME were present from various groups of constituents, such as fitosterols (10.90%) (e.g., β-Sitosterol-5.79%), monoglycerides (9.23%) (e.g., 1-Monopalmitin and 2-linoleoylglycerol), phenols (4.46%) (e.g., 2-Methoxy-4-vinylphenol and 1,2,3-Benzenetriol), carbohydrates (3.17%), phenylpropenoids (2.65%) (e.g., Phenol,2-methoxy-4-(1-propenyl) and cnamic acid), pyrans (1.27%) (e.g., 4H-Pyran-4-one, 2,3-dihydro-3,5-dihydroxy-6-methyl- and maltol), aromatic acids (0.83%) (e.g., benzoic acid and benzeneacetic acid), and other unidentified (NN) compounds (9.44%). Our results agree quite well with those previously published. Munoz and Ramos [34] determined the first quantification of phytosterols in *A. chilensis.* They showed that β-sitosterol was the main sterol component (4.3 ± 1.0 micrograms per gram of dehydrated sample) and identified campesterol, sitostanol, and campestanol. β-sitosterol is probably the most abundant and widely distributed plant sterol, which has promising antidiabetic and antioxidant effects and is used for its cholesterol-lowering properties. Crisóstomo-Ayala et al. [35] described for the first time the quantitative analysis of fatty acids of maqui leaves. The higher values for linoleic and linolenic acid were obtained in the samples of adult leaves presented in ex vitro leaves compared with the in vitro leaves of *A. chilensis.* The highest values of β-sitosterol and α-tocopherol were obtained from the in vitro leaves, followed by spring leaves, and the lowest in winter leaves. In the study contributed by Quispe-Fuentes et al. [6], the major fatty acid in the fresh maqui sample was linoleic acid, accounting for 45.41% of total fatty acids, followed by oleic (34.92%), palmitic (9.49%), stearic (2.92%), and α-linolenic (2.12%) acids. One more author also reported that the major unsaturated acids were linoleic and α-linolenic, while the saturated acids were myristic, palmitic, and stearic acids [29].

Fruits such as maqui have been referred to as “superfruits” due to their phytochemical composition and antioxidant properties [26,29,32]. Due to this, it should be characterized by good quality and safety; in particular, it should be free of all chemical contaminants that pose a risk to consumer health. In this work, the toxic elements level in maqui berry extracts was investigated. As, Cd, and Pb are toxic heavy metals whose accumulation can cause adverse effects on human health due to the mechanism of the production of reactive oxygen species, the appearance of oxidative damage, and autoimmune manifestation [36]. Table 4 contains the concentrations of As, Cd, and Pb in the studied maqui extracts. In our investigation, the highest mean As concentration of 11.83 µg/kg was found in the water extract (MWE) and the lowest, i.e., 1.58 µg/kg, in MEE95. Among the tested samples, the highest mean Cd concentration of 433.33 µg/kg was found in the MWE extract. The concentration of Cd in the ethanol extracts was found to be lower, with 14.31 µg/kg in MEE70 and 7.89 µg/kg in MEE95. In the case of Pb, the average content in the tested extracts ranged from 77.45 µg/kg (MEE70) to 258.81 µg/kg (MWE). According to the European Union Commission Regulation (Commission Regulation (EU) 2023/915), none of the tested elements exceeded the limit of the maximum permissible level [37].

To the best of our knowledge, there has been one study that determined the toxic element levels in maqui berry extracts. Brauch et al. [8] investigated the levels of various toxic metals, including As, Cd, Hg, and Ni. They found that one pollutant toxic to human health, even in microgram quantities, was Ni (0.103–0.315 mg/kg). However, none of the studied toxic elements exceeded the critical levels listed by the European Food Safety Authority (EFSA) and the Food and Drug Administration (FDA).

### 2.3. Cell Viability, DNA Synthesis, and Photoprotective Role

**Figure 1:**
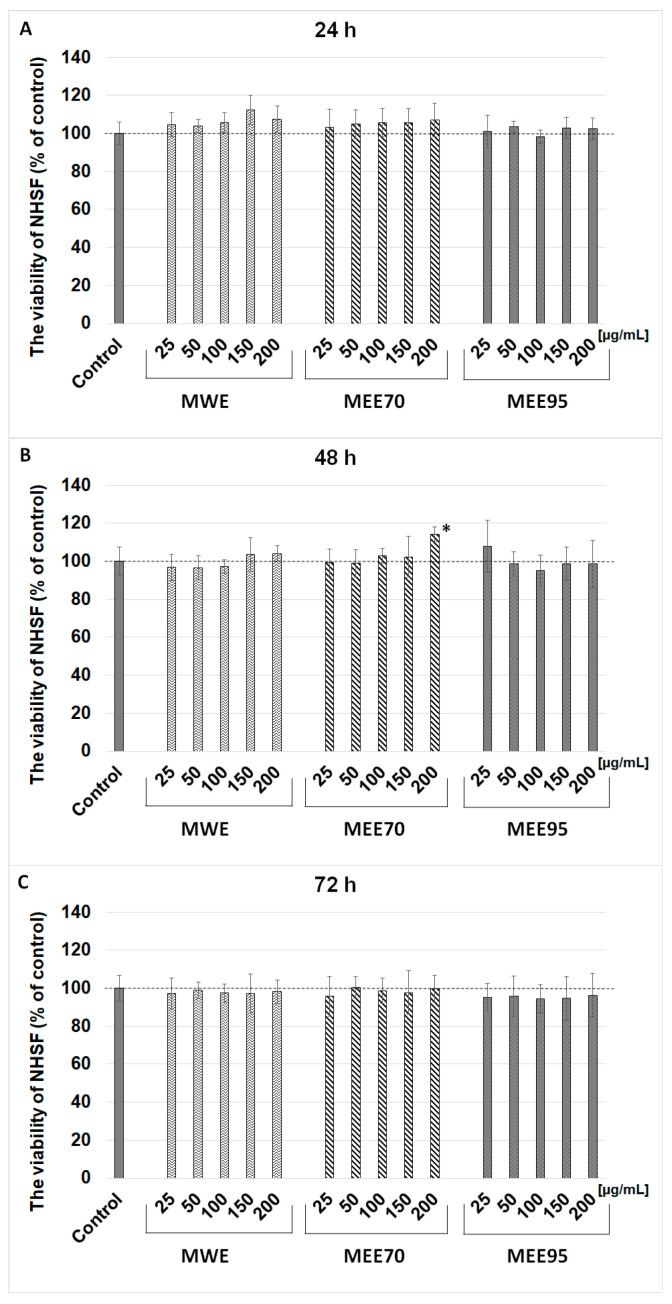
Effects of MWE, MEE70, and MEE95 extracts at different concentrations (25, 50, 100, 150, 200 µg/mL) on the viability of normal human skin fibroblasts (NHSF) after 24 (**A**), 48 (**B**), and 72 h (**C**) of incubation. Results are presented as percentage of control. * *p* < 0.05—statistically significant difference from control.

**Figure 2:**
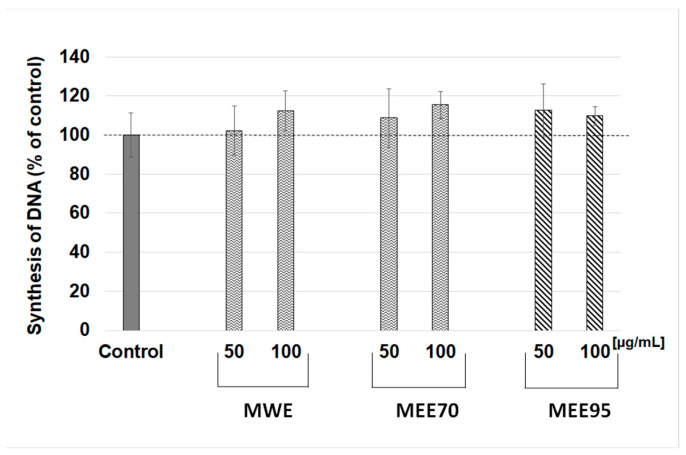
Effect of MWE, MEE70, and MEE95 extracts at different concentrations (50, 100 µg/mL) on the incorporation of [3H]-thymidine into DNA of normal human skin fibroblasts NHSF cells after 48 h incubation. Results are presented as percentage of control.

**Figure 3:**
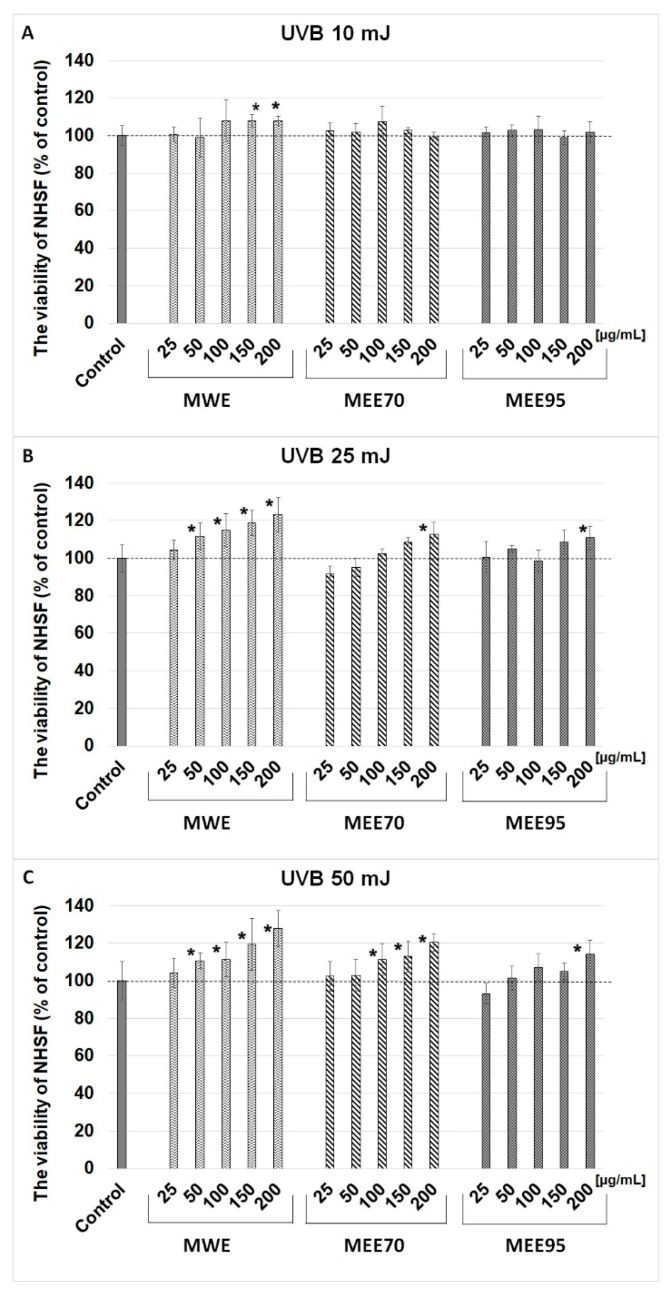
Effect of MWE, MEE70, and MEE95 extracts at different concentrations (25, 50, 100, 150, 200 µg/mL) on viability of NHSF cells after application of UV radiation ((**A**) 10 mJ/cm^2^, (**B**) 25 mJ/cm^2^, (**C**) 50 mJ/cm^2^). Results are presented as percentage of control. * *p* < 0.05—statistically significant difference from control.

Ultraviolet B (UVB) irradiation induces DNA damage, oxidative stress, inflammatory processes, accelerated skin aging, and photocarcinogenesis [22]. The use of phytochemicals with antioxidant properties is one of the possibilities for protecting skin against the dangerous effects of UV radiation. Berries, such as maqui, currants, and blueberries, are rich in bioactive compounds, such as polyphenols, which can act as chemoprotectants. Maqui berry (*Aristotelia chilensis*) is a natural antioxidant, anticancer, and anti-inflammatory food. In the present work, we studied the potential photoprotective capacity of maqui berry extracts and to the best of our knowledge, it is the first research that evaluated maqui in terms of protective and restorative effects against UV-treated human skin fibroblast cells. In the first stage, the possible cytotoxic effects of the maqui extracts were studied depending on their increasing concentration and incubation time. Based on the in vitro MTT assay, the viability of human skin fibroblast cells treated with extracts from maqui berry (MWE, MEE70, MEE95) at concentrations from 25 to 200 µg/mL was estimated (Figure 1). The tested extracts of maqui berry did not inhibit the viability of NHSF cells after 24, 48, and 72 h of incubation. The viability ranged from 94.3 ± 7.4 to 114.1 ± 3.8% vs. control and only a dose of 200 µg/mL of MEE70 extract significantly increased the viability after 48 h of incubation. The assessment of cell proliferation was based on the level of [^3^H]-thymidine incorporation during DNA synthesis on fibroblast cells treated with MWE, MEE70, and MEE95 extracts at doses of 50 and 100 µg/mL. The research showed that the MWE and MEE70 extracts at a dose of 100 µg/mL resulted in increased DNA syntheses to 112.3 ± 10.3% and 115.4 ± 7.0%, respectively, but statistical significance was not observed (Figure 2). The NHSF cells were treated with three doses of UVB radiation (10, 25, and 50 mJ/cm^2^) to study the photoprotective effect of maqui berry extracts. The results show that the MWE extract treatment (doses of 150–200 µg/mL (Figure 3A) and 50–200 µg/mL (Figure 3B,C)) resulted in a significant (*p* > 0.05) and dose-dependent increase in NHSF cells viability after the application of UV radiation. The increased cell viability ranged from 8% to 27%. The use of MEE70 extract (200 µg/mL–25 mJ/cm^2^ radiation and 100–200 µg/mL–50 mJ/cm^2^ radiation) showed a significant increase in cell viability up to 13% and 11–20% (Figure 3B,C). The MEE95 extract significantly influenced the NHSF viability at the 200 µg/mL dose (after application of 25 and 50 mJ radiation), where the increases in cell viability were about 10 and 14% (Figure 3B,C).

There was only one previous study that showed the photoprotective properties of maqui berry extract, which was determined using human keratinocytes [23]. The authors reported that MEE reversed the DNA damage induced by UVB irradiation in HaCaT cells by upregulating endogenous cellular enzymatic and non-enzymatic antioxidant systems containing superoxide dismutase, catalase, and glutathione and reducing the production of nitric oxide. It was also shown that maqui berry extract treatment enhanced the antioxidant ability and weakened lipid peroxidation in BALB/c mice exposed to UVB radiation, downregulated interleukin (IL)-6 and tumor necrosis factor-α levels, and upregulated IL-4 levels. Another study [24] investigated the protective effects of maqui extracts and its major anthocyanins (delphinidin 3,5-O-diglucoside (D3G5G) and delphinidin 3-O-sambubioside-5-Oglucoside (D3S5G)) against light-induced murine photoreceptor cell death. These authors found that maqui extracts and their anthocyanidins inhibit light-induced photoreceptor cell death due to suppressed ROS production. They also suggested that the inhibition of phosphorylated- p38 (MAPK, ERK; mitogen-activated protein kinases) may be involved in the underlying mechanism. It was shown that the phosphorylation of MAPKs (ERK, p38) activated by UVB irradiation is inhibited by treatment with galangin in a dose-dependent manner. When the MAPK signaling pathway is activated by UVB stimulation, the MAPK protein phosphorylates the heterodimers c-Jun and c-Fos of transcription factor AP-1 and NF-kB to upregulate MMPs [38]. Furthermore, research conducted by Bae et al. [39] demonstrated that blueberry (*Vaccinium uliginosum* L.) anthocyanins have potential in UV-B-induced skin photoaging by blocking collagen destruction and inflammatory responses through NF-κB transcriptional mechanisms and MAPK signaling. Lecci et al. [40] showed that polyphenol fractions from olive mill wastewater exerted an effective antioxidant activity in vitro and in cells when administered together with UV-radiation on HEKa (human epidermal keratinocytes adult). Pro-oxidative and pro-apoptotic effects on the UVA-damaged HEKa cells were observed, suggesting some protective actions of the polyphenol fraction on keratinocyte cell cultures.

This work provides the basis for more advanced research to test the effectiveness of maqui as a protective factor against UV radiation. Further studies are required to determine which components of the extracts show a photoprotective effect.

## 3. Materials and Methods

### 3.1. Reagents and Solutions

Dulbecco’s modified eagle medium (DMEM), fetal bovine serum (FBS), trypsin-EDTA, penicillin, and streptomycin were purchased from Gibco (Thermo Fisher Scientific, Waltham, MA, USA).

Calcium-free phosphate-buffered saline (PBS) was received from Biomed (Lublin, Poland). Bis(trimethylsilyl)trifluoroacetamide (BSTFA) with an addition of 1% trimethylchlorosilane, C_10_–C_40_ n-alkane standard solution, methylthiazolyl diphenyl-tetrazolium bromide (MTT), dimethyl sulfoxide (DMSO), pyridine, trichloroacetic acid, hydrochloric acid and a trizma base, and a matrix modifier (0.5% ammonium dihydrogen phosphate for Cd and Pb determination) were obtained from Sigma-Aldrich (St. Louis, MO, USA).

Ethanol at 95% was obtained from AWW Group, Poland. The scintillation cocktail was purchased from PerkinElmer (Boston, MA, USA) and methyl-3H thymidine from MP Biomedicals, Inc. (Irvine, CA, USA).

Analytical grade chemicals and ultrapure water were used throughout the procedure. The ultrapure water necessary for all tests was prepared with Simplicity 185 (Millipore, Burlington, NJ, USA), which is a system of water purification.

The certified reference material (Mixed Polish Herbs, Institute of Nuclear Chemistry and Technology, Warsaw, Poland) was used for the quality control.

The NexION Setup Solution (Perkin Elmer, Boston, MA, USA) was used for the ICP-MS optimization.

The following reagents were used to assess the antioxidant properties: Folin–Ciocalteu reagent Na_2_CO_3_; Gallic acid; 2,2-diphenyl-1-picrylhydrazyl–DPPH; Trolox; stock solutions of 1000 mg/L served for the preparation of standard solutions of As, Cd, and Pb; and spectrally concentrated ultrapure nitric acid 69% (69% HNO_3)_ were used in the acid digestion step (Merck, Darmstadt, Germany).

### 3.2. Material and Extracts Preparation

Maqui berry powder made from dried berries (Chilean origin) was purchased from a local market. A portion of maqui fruit was extracted using different solvent systems: ethanol 70% (EtOH70%), ethanol 95% (EtOH95%), water for cell culture, and methanol (MeOH) for the GC-MS analysis. To prepare maqui EtOH extracts (MEE), the 100 g of maqui powder was soaked in 900 g of 70% (MEE70) or 95% (MEE95) ethanol (1:10) for 24 h and then extracted using a supersonic washer for 30 min. The extracts were centrifuged within 10 min at approximately 3000 rpm to separate the pellet and liquid phases. The remaining sediment was soaked with half of the portion of ethanol (450 g), and then processed again as reported above. The remaining supernatants were mixed in a round-bottom flask and evaporated using a Rotavapor R-100 rotary evaporator (Büchi, Flawil, Switzerland) under a reduced pressure of 10 ± 2 mbar at 35 °C. The samples were frozen and lyophilized until dryness. For the water extraction, 100 g of the powder material was mixed with 900 g of water for 3 h at 80 °C, repeatedly extracted three times, and filtered to prepare water extract. Then, the extract was freeze dried to powder for 72 h.

### 3.3. Determination of Antioxidant Capacity Using DPPH Assays

The free radical scavenging capacity was performed according to the method described by Sánchez-Moreno et al. [41] with our own modification using an ethanol solution of DPPH at a concentration of 0.1 mmol/L.

This method is widely used to test the antioxidant capacity of fruits, vegetables, and juices. To perform the analysis, 0.1 mL of the sample was added to 2.9 mL of the DPPH solution and mixed. Absorbance was measured on a Hitachi U-2001 spectrophotometer (Hitachi, Tokyo, Japan) at 517 nm after 30 min of incubation in the dark at room temperature. The free radical scavenging percentage was calculated using the formula

DPPH [%] = [1 − Ax/A0] × 100,

where Ax is the absorbance for the sample solution and A0 is the absorbance of the control (the sample solution before incubation). In addition, the result was expressed in mg Tx/L.

### 3.4. Determination of Total Phenolic Content (TPC) Analysis

Powder total polyphenol content (TPC) was evaluated using the Folin–Ciocalteu method in accordance with the procedure described by Sarkis et al. [42], which was modified. To the TPC extract, 0.5 g of powder was dissolved in 10 mL water and shaken in a vortexer (Heathrow Scientific Vortexer, Vernon Hill, IL, USA) at 2000 rpm for 5 min and the supernatant was immediately collected in a Falcon tube. The following were added to a test tube: 250 μL supernatant, 1250 μL Folin–Ciocalteu reagent, and 1000 μL 7.5% anhydrous sodium carbonate; the test tube was shaken and left in darkness for 120 min, after which an absorbance reading was taken at 760 nm with a spectrophotometer (UV-VIS, Hitachi U-2001, Tokyo, Japan). Gallic acid solutions between 0 and 1000 μL/g were used to construct the calibration curve. The results were expressed in mg of gallic acid equivalents per gram of sample (mg GAE/g).

### 3.5. Determination of Toxic Metal Levels—As, Cd, and Pb

The levels of toxic elements were investigated using inductively coupled plasma–mass spectrometry (ICP-MS, NexION 300D, Perkin Elmer, Waltham, MA, USA). To determine the concentration of As, Cd, and Pb, maqui berry extract samples were homogenized in a stainless steel mill before digestion, weighed (0.2–0.3 g), and placed in mineralization polytetrafluoroethylene vessels. Then, 4 mL of spectrally pure concentrated (69%) HNO_3_ was added (Tracepur, Merck, Darmstadt, Germany). Microwave digestion was performed in a closed-loop system (Berghof, Speedwave, Eningen, Germany). The mineralization process included four steps with different temperatures (1st—170 °C, 2nd—190 °C, 3rd—210 °C, 4th—50 °C) and pressures (1st—20 atm, 2nd—30 atm, 3rd—40 atm, 4th—40 atm) for 10, 10, 10, and 18 min, respectively. The overall process consisted of four steps, presented in Table 5. After mineralization, the samples were quantitatively transferred to polypropylene vessels and then diluted 10 times. A kinetic energy discrimination (KED) chamber was used in the case of As, and in the standard mode in the cases of Cd and Pb. To correct for polyatomic interference in this configuration, kinetic energy discriminations and collisions were used. The results were obtained in counts per second (cps) and based on calibration curves; then, they were converted into concentrations. To determine the limit of detection (LOD), 10 independent blank determinations were made. A three-fold standard deviation (SD) from the mean value determined in concentration units was taken as the LOD. The LOD values were 0.018 μg/kg for As, 0.017 μg/kg for Cd, and 0.16 μg/kg for Pb.

The contents of As, Cd, and Pb were calculated and are shown as µg/kg of product. A detailed description of the ICP-MS parameters for the As, Cd, and Pb determinations is presented in Table 6. The results of the toxic metal levels were compared with the norms given for food supplements according to the European Union Commission Regulation (Commission Regulation (Eu) 2023/915) Commission Regulation (EU) 2023/915 of 25 April 2023 on maximum levels for certain contaminants in food and repealing Regulation (EC) No 1881/2006 [37]. The maximum permissible cadmium level in food supplements is 1000 µg/kg; 3000 µg/kg for lead; and for arsenic, there are no direct maximum levels. For example, the maximum level of As in fruit juices, concentrated fruit juices as reconstituted, and fruit nectar is 20 µg/kg.

Quality control was performed by analyzing the certified reference material (Mixed Polish Herbs, Institute of Nuclear Chemistry and Technology, Warsaw, Poland) prior to the start of the analysis. The reference material was analyzed for every tenth studied sample. The results of the quality control are summarized in Table 7.

### 3.6. Gas Chromatography–Mass Spectrometry (GC-MS) Analysis

To prepare the maqui methanol extract (MME), 6 g of maqui dry powder was mixed with methanol and water (80:20), acidified with 1 M HCl to pH 2 solution, and extracted for 5 min at 40 °C in an ultrasonic bath. The supernatant was decanted and centrifuged. The residues were mixed with an acetone and water (70:30) solution and sonicated (5 min, 40 °C). The supernatants were combined, filtered through a filter paper, and then evaporated on the evaporator. The residue was taken up with 20 mL of distilled water and purified on conditioned SPE disks. The extract was washed with 1% methanol (40 mL), dried with calcium carbonate, and allowed to evaporate. A total of 5 mg of MME was diluted with 220 μL of pyridine and 80 μL of BSTFA with an addition of 1% trimethylchlorosilane. The reaction mixture was sealed and heated for 0.5 h at 60 °C to form trimethylsilyl (TMS) derivatives.

GC-MS analysis of MME was performed using a Clarus 680 gas chromatograph with a Clarus 600 T MS mass selective detector (PerkinElmer, Walthman, MA, USA) equipped with an Elie-5 MS fused silica column (30 m, 0.25 mm i.d., 0.25 lm film thickness), with electronic pressure control and a split/splitless injector. The helium flow rate through the column was 1 mL/min in a constant flow mode. The injector worked at 250 °C in the split (1:50) mode. The initial column temperature was 50 °C, rising to 310 °C at 5 °C/min, and the higher temperature was maintained for 15 min. The MSD detector acquisition parameters were as follows: transfer line temperature 280 °C, MS source temperature 230 °C, and MS quad temperature 150 °C. The EIMS spectra were obtained at the ionization energy of 70 eV. The MSD was set to scan 41–600 amu. Following the integration, the fraction of each component in the total ion current was calculated. Hexane solutions of C_10_–C_40_ n-alkanes were separated under the above conditions. Gas chromatographic linear programmed retention indices (ITs) were calculated based on the retention times of the n-alkanes hexane solution and separated components of the extract samples.

To identify the separated components, two independent analytical parameters were used: mass spectra and calculated retention indices. The mass spectrometric identification of non-derivatized components was performed with an automatic system for GC-MS data processing supplied by the NIST 14 library (NIST/EPA/NIH Library of Electron Ionization Mass Spectra). The mass spectra and retention indices of the components registered in the form of TMS derivatives were compared with those presented in a previously published database [43] and a private mass spectra library. Identification was considered reliable if the results of the computer search of the mass spectra library were confirmed using experimental RI values, i.e., if their deviation from the published database values did not exceed ± 10 u.i. (the average quantity of inter-laboratory deviation for non-polar stationary phases).

### 3.7. Cell Culture

A normal human skin fibroblast (NHSF) cell line (CRL-1474) was obtained from the ATCC collection (Rockville, MD, USA). The cells were cultured in a humidified incubator (Binder, Tuttlingen, Germany) at 37 °C and 5% CO_2_ atmosphere to obtain confluence in Dulbecco’s Modified Eagle Medium (DMEM) (Gibco, Waltham, MA, USA) with the addition of 10% Fetal Bovine Serum (FBS) (Gibco, Waltham, MA, USA) and 1% solution of Penicillin-Streptomycin (Sigma, Burlington, MA, USA).

#### Cytotoxicity Assay

Cell viability was measured using an MTT assay. The effects of maqui extracts (MEE70, MEE95, MWE) on NHSF were studied after 24 h, 48 h, and 72 h of the treatment. Cells at a density of 2 × 10^4^ cells/mL were seeded onto 96-well plates at a volume of 200 µL per well. The plates were incubated for 24 h at 37 °C and 5% CO_2_ to obtain confluence. To achieve appropriate dilutions of the tested extracts (25, 50, 100, 150, and 200 µg/mL), “STOCK” solutions with a concentration of 1000 µg/mL were prepared. We dissolved 10 mg of freeze-dried extracts in DMSO (Dimethyl Sulfoxide) (Sigma, Burlington, MA, USA) and then transferred them to DMEM. To maintain constant conditions during the experiment, both the medium (control) and solutions were replaced after 24 h. After 48 h, the medium was removed, the cells were rinsed with 200 mL of PBS (Sigma, Burlington, MA, USA), and MTT (3-[4,5-dimethylthiazol-2-yl]-2,5-diphenyl tetrazolium bromide) (Sigma, Burlington, MA, USA) dissolved in PBS (5 mg/mL) was added to the wells. After 2 h of incubation, the MTT solution was removed. The formed formazan was dissolved by adding 180 µg/L DMSO to each cavity and 20 µg/L Sorensen buffer. The absorbance was read using a Multimode Plate Reader Victor X3 (PerkinElmer, Singapore) at 570 nm and viability was shown as a percentage of the control (medium with 0.1% DMSO).

### 3.8. DNA Synthesis Assay

DNA biosynthesis in the NHSF cell line after the treatment of maqui berry extracts was carried out using a radioisotope method by measuring the incorporation of [^3^H]-thymidine into the cells’ DNA. Based on the results of the MTT test, maqui extracts at concentrations of 50 and 100 µg/mL were selected for thymidine incorporation. NHSF cells were seeded (1.5 × 10^5^ cell/well) on 24-well plates in DMEM supplemented with 10% heat-inactivated FBS, 100 U/mL penicillin, and 0.1 mg/mL streptomycin and exposed to the treatment medium containing DMSO (0.1%—control). After obtaining 80–85% confluence, the cells were treated with maqui extracts dissolved in DMEM with inactivated FBS and incubated for 44 h. Next, 10 µL [^3^H]-thymidine was added to each well and incubated again for 4 h. After that time, the medium was removed, and the cell surface was washed twice with 1 mL of 0.05 M cold tris(hydroxymethyl)aminomethane hydrochloride (Tris-HCl) and 1 mL of 5% trichloroacetic acid (TCA). A total of 1 mL of sodium dodecyl sulfate (SDS) was added to each well, and cell lysate was transferred to the vessels with scintillator fluid. The level of [^3^H]-thymidine incorporated in the newly synthesized DNA strand was assessed using a scintillation counter TriCarb 2810 TR (PerkinElmer, Singapore) in relation to the number of cells proliferating during the S phase of the cell cycle.

### 3.9. UVB Irradiation

The effect of maqui berry extracts on the NHSF cells exposed to UVB radiation was assessed after irradiation with UVB (10, 25, and 50 mJ/cm^2^) before evaluating the DNA damage. Cells at a density of 2 × 104 cells/mL were seeded onto 96-well plates at a volume of 200 µL per well. The plates were incubated for 24 h at 37 °C and 5% CO_2_ to obtain confluence. To achieve appropriate dilutions of tested extracts (25, 50, 100, 150, and 200 µg/mL), “STOCK” solutions with a concentration of 1000 µg/mL were prepared. Next, the cells were incubated with extract for 24 h. Before the UVB exposure, the cells were gently washed three times with PBS and then covered with a thin glass layer. Control cells with no irradiation were treated similarly and stored in the dark. The irradiation intensity was detected using a UVB radiometer Crosslinker CL-100 (UVP Analytik Jena, Upland, CA, USA). The PBS layer was removed from the plates and fresh medium or the medium with extracts was added and incubated. Then, the incubation MTT test was performed. The results were also compared with the control without the extracts.

### 3.10. Statistical Analysis

Statistical analyses were performed using Statistica v.13 software. Differences between independent and dependent groups were tested using the nonparametric Mann–Whitney U-test. The correlations were calculated and tested using the Spearman rank test. A *p*-value of <0.05 was considered significant.

## 4. Conclusions

The results of the current study suggest that maqui extracts could be a good source of antioxidants and lipophilic compounds for nutraceutical and pharmaceutical industries. Maqui berry extracts have a slightly protective effect against skin damage caused by UVB radiation. These are preliminary studies that require further research to determine which maqui compounds correspond with the photoprotective activity.

## Figures and Tables

**Table 1:** Characteristics of the antioxidant properties of the studied extracts.

Type of Extract	DPPH (mg Tx/L)	DPPH (% of Free Radical Scavenging)	TPC (mg GAE/100 g)
MWE	283.63 ± 7.29	66.04	57.75 ± 0.44
MEE70	284.60 ± 4.31	64.35	56.47 ± 0.19
MEE95	211.80 ± 6.14	46.48	56.65 ± 0.69

MWE—maqui water extract; MEE70—maqui 70% ethanol extract; MEE95—maqui 95% ethanol extract; TPC—total polyphenol content.

**Table 2:** The main compounds groups in chemical composition of the maqui methanol extract.

Group of Compounds	MME (%)
Unsaturated fatty acids and esters	34.77
Saturated fatty acids and esters	23.18
Fitosterols	10.90
Monoglycerides	9.23
Phenols	4.56
Carbohydrates	3.17
Phenylpropenoids	2.65
Pyrans	1.27
Aromatic Acids	0.83
Other compounds	9.44
Sum	100

**Table 3:** Chemical composition of the maqui extract (MME).

Components, TMS Derivative	Rt (min)	LTPRI ^Exp^	LTPRI ^Lit^	Relative Composition (%)
N,N-73, 75, 117, 103	12.77	1032	-	0.92
N,N-97, 68, 98, 42	16.44	1108		0.38
4H-Pyran-4-one, 2,3-dihydro-3,5-dihydroxy-6-methyl-	17.74	1141	1151	0.89
Maltol	20.90	1210	1208	0.39
N,N-120, 174, 91, 65	21.42	1221	-	0.11
5-Hydroxymethylfurfural	21.78	1230	1233	3.55
Benzoic Acid	22.59	1248	1249	0.34
Octanoic acid	23.53	1266	1266	0.22
Benzeneacetic acid	24.83	1298	1302	0.37
2-Methoxy-4-vinylphenol	25.66	1317	1317	1.11
Nonanoic acid	28.01	1363	1355	2.50
1,2,3-Benzenetriol	28.36	1381	1386	3.10
Phenol, 2-methoxy-4-(1-propenyl)-	31.27	1451	1450	1.87
Decanoic acid	31.67	1461	1460	0.13
β-D-Glucopyranose, 1,6-anhydro-	32.77	1488	1487	2.68
N,N-73, 129, 75, 145	33.00	1493	-	1.64
N,N-73, 75, 117, 129	33.35	1503	-	1.24
N,N-73, 239, 44. 75	34.04	1520	-	0.29
Cinnamic acid	34.98	1550	1542	0.30
4-Hydroxybenzoic acid	38.38	1635	1635	0.13
Ethanone, 1-[4-(methylsulfonyl)phenyl]-	38.75	1645	1649	0.12
Homovanillic acid	38.94	1650	1658	0.26
Dodecanoic acid	39.23	1658	1655	0.22
Levoglucosan	41.87	1710	1694	0.19
(E)-Coniferyl alcohol	42.15	1740	1743	0.48
Myristic acid	44.12	1796	1794	0.22
D-(-)-Tagatofuranose, pentakis(trimethylsilyl) ether (isomer 1)	44.27	1801	1801	0.30
Protocatechoic acid	45.47	1836	1835	0.09
Tetradecanoic acid	46.06	1854	1850	0.46
N, N-73, 310, 44, 254	46.61	1870		0.31
Hexadecanoic acid, methyl ester	48.46	1928	1926	0.50
Pentadecanoic acid	49.24	1952	1950	0.11
Hexadecanoic acid, ethyl ester	50.63	1996	1993	1.98
9-Hexadecenoic acid, (Z)-	51.64	2029	2027	0.29
Palmitic Acid	52.33	2052	2050	14.92
Linoleic acid, methyl ester	53.65	2096	2092	0.57
6-Octadecenoic acid, methyl ester, (Z)-	53.83	2102	2105	0.69
cis-Vaccenic acid	55.02	2143	2139	2.51
Linoleic acid ethyl ester	55.63	2164	2162	4.31
Oleic acid, ethyl ester	55.81	2170	2173	3.83
Octadecanoic acid, ethyl ester	56.58	2196	2195	0.28
9,12-Octadecadienoic acid (Z,Z)	57.12	2216	2212	7.49
Oleic Acid, (Z)	57.29	2222	2218	14.17
13-Octadecenoic acid	57.46	2228	2228	0.96
Stearic acid	58.02	2248	2246	1.65
N, N-103, 73, 44, 131	66.41	2568	-	0.81
1-Monopalmitin	67.36	2607	2607	0.14
2-linoleoylglycerol	70.47	2739	2739	7.33
1-Monooleoylglycerol	71.25	2772	2784	1.77
β-Sitosterol	82.57	3214	3200	5.79
β-Sitosterol	83.23	3334	3344	5.13
Sum	-	-	-	100

LTPRI—linear temperature programmed retention index, Rt—retention time.

**Table 4:** Content of toxic elements (As, Cd, Pb) in the tested maqui berry extracts compared with maximum limits.

	As		Cd		Pb	
Type of Extract	As (µg/kg)	Norm *	Cd (µg/kg)	Norm ** (µg/kg)	Pb (µg/kg)	Norm ** (µg/kg)
MWE	11.83	-	433.33	1000	258.81	3000
MEE70	3.37		14.31		77.45	
MEE95	1.58		7.89		78.38	

* No available norm for the food supplements, ** Commission Regulation (EU) 2023/915-norm for the food supplements.

**Table 5:** Steps and parameters of microwave digestion of Maqui berry powder in a closed-loop system (Berghof, Speedwave, Eningen, Germany).

Phase	Temperature (°C)	Pressure (atm)	Time (min)	Power (%)
I	170	20	10	90
II	190	30	10	90
III	210	40	10	90
IV	50	40	18	0

**Table 6:** ICP-MS conditions for As, Cd, and Pb determinations.

Parameter	Analytical Conditions		
	As	Cd	Pb
Mode	KED	Standard	Standard
Mass (amu)	75	110 111 113 114	206 207 208
Dwell time per amu (ms)	50	50	50
Integration time (ms)	1000	1000	1000
Detector calibration mode	Dual	Dual	Dual
Replicants	5	5	5

ICP-MS—inductively coupled plasma–mass spectrometry, KED—kinetic energy discrimination.

**Table 7:** Results obtained in the quality control process.

Element	Precision (%)	Recovery (%)	Declared Concentration in CRM (µg/kg)
As	3.3	99.0	10
Cd	2.5	99.1	7
Pb	2.4	99.5	52

CRM—certified reference material.

## Data Availability

The data presented in this study are available on request from the corresponding author.
